# The relationship between adiposity and bone density in U.S. children and adolescents

**DOI:** 10.1371/journal.pone.0181587

**Published:** 2017-07-19

**Authors:** Cecilia Gállego Suárez, Benjamin H. Singer, Achamyeleh Gebremariam, Joyce M. Lee, Kanakadurga Singer

**Affiliations:** 1 Department of Pediatrics and Communicable Diseases, Division of Pediatric Endocrinology and Metabolism, University of Michigan Medical School, Ann Arbor, Michigan, United States of America; 2 Department of Internal Medicine, Division of Pulmonary and Critical Care Medicine, University of Michigan Medical School, Ann Arbor, Michigan, United States of America; 3 Child Health Evaluation and Research Unit (CHEAR), Department of Pediatrics and Communicable Diseases. University of Michigan Medical School, Ann Arbor, Michigan, United States of America; University of Arkansas for Medical Sciences College of Pharmacy, UNITED STATES

## Abstract

**Objective:**

In adults, obesity has been associated with several health outcomes including increased bone density. Our objective was to evaluate the association between percent body fat and fat mass with bone mineral density (BMD) in a nationally representative population of children and adolescents.

**Study design:**

A total of 8,348 participants 8–18 years of age from the National Health and Nutrition Examination Survey (NHANES) 1999–2006 had whole body DXA scans performed. We conducted linear regressions to examine the relationship between percent body fat and fat mass with outcome variables of total body, pelvic and lumbar spine areal BMD (aBMD), controlling for lean body mass and assessing for gender and race/ethnicity interactions.

**Results:**

We found evidence of gender and race/ethnicity interactions with percent body fat and total fat mass for the different BMD areas. Generally, there were decreases in total body aBMD (p<0.001) and lumbar spine aBMD (p<0.001) with increasing percent body fat and total fat mass, with less consistent patterns for pelvic aBMD.

**Conclusion:**

Our findings of regional differences in the relationship of adiposity to aBMD in children and adolescents with significant interactions by gender and race/ethnicity emphasizes the need for further investigations to understand the impact of adiposity on bone health outcomes.

## Introduction

Obesity and its related medical diseases continue to be a significant problem in both adults and children. Currently 31.8 percent of children aged 2 to 19 years in the U.S. are categorized as overweight or obese[1] and there are many consequences of this excess weight in children, including hypertension [2, 3], type 2 diabetes [4], sleep apnea, hyperlipidemia, and increased risk for immune diseases [5]. The effects of excess weight on bone health are less well studied, but since childhood and adolescence are critical stages for skeletal mineralization, it is important to understand how body composition during this period may influence bone mineralization and may affect bone health.

The majority of clinical studies evaluating the relationship between weight status and bone mineral density (BMD) have been performed in adult populations, which have shown a positive association of body mass index (BMI) with total body BMD [6]. Pediatric studies have reported contradictory results, with some reporting a negative association of visceral adipose tissue and BMD [7], and percent body fat with BMD and/or bone mineral content (BMC) [8], while others report a positive association of BMI on BMD [9] and BMC [8]. This same association has been suggested to not be the case for children who have a BMI greater than the 95^th^ percentile [10]. Limitations of these studies include small sample sizes, and the use of BMI as a measure of body fat, which may not accurately categorize children with increased lean mass [11]. BMI also does not correlate with body fat similarly across ethnic and racial groups [11–13], or genders [14].

Few studies have looked at the association of fat mass on bone density in children and adolescents while adjusting for lean body mass [15]. Given that lean mass is a major contributor to BMI/weight status and has a direct mechanical impact on the bone that contributes to an increase in BMD [16], it is important to control for lean mass when investigating the relationship between fat mass and bone mineral density. The objective of our study was to examine associations between percent body fat and total fat mass on total body, pelvic and lumbar spine aBMD in a nationally representative sample of U.S. children and adolescents, and to examine gender and race/ethnicity interactions in these associations. We hypothesized that higher percent body fat would be associated with lower aBMD across all regions.

## Materials and methods

We used data from National Health and Nutrition Examination Survey (NHANES) 1999–2006, a nationally representative cross-sectional survey of adults and children in the US civilian non-institutionalized population which oversamples individuals 12–19 years of age and minority populations and collects survey information about health, anthropometric measures, and laboratory data [17].

NHANES uses a complex, multistage, probability design in order to select representative participants [18]. It provides weight, height, gender, age, and race/ethnicity for each participant. As the survey reports, we used race/ethnicity classification as following: 1) White/non-Hispanic, 2) African American/non-Hispanic, 3) Hispanic, 4) Other races (which includes all non-Hispanic persons reporting a race other than White or African American, all participants who reported multiple races and the non-Hispanic Asians participants as well). NHANES performed whole body DXA scans in a subset of individuals 8 years and older using a Hologic QDR 4500 fan-beam densitometer (Hologic, Inc., Bedford, Massachusetts). Scans were conducted in the fast mode by trained radiology technologists and were analyzed using Hologic DOS software version 8.26:a3. Detailed documentation of the procedures for the DXA scans are available online [17, 19]. The NHANES DXA scan data provides bone and soft tissue measurements of total body, arms, legs, trunk and head; bone measurements from whole body DXA scans (total body, pelvic, left and right ribs, thoracic spine, lumbar spine); and values for the total body and bone regions including total mass (grams), Bone Mineral Content (BMC) (grams), bone area (cm^2^), areal bone mineral density (aBMD) (gm/cm^2^), fat mass (gm), lean mass excluding BMC (gm), lean mass including BMD (gm) and percent body fat [19]. NHANES provides the DXA data as multiply imputed datasets because of nonrandom missing data [17, 19].

Similar to our previous study [20], we calculated sex- and age-specific percentiles of body fat based on the literature [11, 20]. We excluded study participants with sex- and age-specific percentiles less than the 5^th^ percentile, because these individuals could have other underlying disease or illness.

Our dependent variables were total body, pelvis and lumbar spine aBMD (g/cm^2^), which were chosen based on previous studies that have evaluated bone density in these areas [8, 15, 21]. Our primary independent variables were percent body fat and fat mass (in kg). Covariates included lean mass (kg), age, gender and race/ethnicity.

### Statistical analysis

We performed separate multiple linear regressions to examine the relationship between percent body fat and total body, pelvic, and lumbar spine aBMD, adjusting for lean mass, age, gender and race/ethnicity. In an additional set of models, we performed separate multiple linear regressions to examine the relationship between total fat mass and total body aBMD, pelvic aBMD and lumbar spine aBMD, adjusting for lean mass, age, gender and race/ethnicity. We first examined 3-way interactions effects of fat variables x gender x race/ethnicity. All 3-way interactions were not significant except for the interaction of total fat mass predicting lumbar spine aBMD. We then examined 2-way interactions of percent body fat (percent body fat x gender; percent body fat x race/ethnicity) with each aBMD region, and total fat mass (total fat mass x gender; total fat mass x race/ethnicity) with each aBMD region, and only show results for models with significant interactions.

To illustrate the associations and interactions, we generated predicted outcome values for total body, pelvic and lumbar spine aBMD using selected percent body fat (or total fat mass) values for each gender and race/ethnicity while fixing all other predictor values at their mean. All statistical analyses account for the complex sampling design in NHANES using sample weights and sample design variables provided by NHANES[22]. We used STATA 13 statistical software for all analysis, which incorporates appropriate sampling weights to adjust for the complex sample design. A p-value threshold of 0.05 was considered to be significant.

## Results

Of 9,797 individuals aged 8–18 years, we excluded children with incomplete DXA scans (n = 911), missing BMI (n = 35), missing age-, and with sex-adjusted percentile body fat less than the 5^th^ percentile (n = 503), which left a sample size of 8,348 (Table 1).

**Table 1:** Demographic characteristics of the included population.

						Male				Female			
weighted% (unweighted n)	Overall Population	White	African American	Hispanic	Other *	White	African American	Hispanic	Other	White	African American	Hispanic	Other *
**Overall**	8348	62.2% (2271)	13.6% (2548)	17.8% (3183)	6.5% (346)	57.2% (1270)	55.9% (1436)	57.5% (1848)	59.5% (191)	42.8% (1001)	44.1% (1112)	42.5% (1335)	40.1% (155)
**Male**	57.3% (4745)												
**Body Fat Percentile**													
**5–70**	69.3% (5583)	71.3% (1619)	71.0% (1823)	59.6% (1898)	73.4% (243)	70.4% (897)	73.9% (1070)	60.2% (1093)	71.6% (131)	72.6% (722)	67.3% (753)	58.9% (805)	75.9% (112)
**70–85**	15.2% (1338)	14.3% (324)	13.3% (329)	20.0% (631)	15.0% (54)	15.7% (196)	12.2% (168)	18.8% (365)	15.2% (31)	12.4% (128)	14.6% (161)	21.6% (266)	14.7% (23)
**85–90**	5.8% (509)	5.7% (127)	5.2% (135)	6.9% (228)	4.7% (18)	5.3% (67)	4.6% (66)	8.1% (139)	4.7% (10)	6.2% (60)	6.1% (69)	5.2% (89)	4.7% (8)
**> 90**	9.7% (918)	8.7% (201)	10.5% (261)	13.5% (426)	7% (31)	8.6% (110)	9.4% (132)	12.9% (251)	8.4% (19)	8.8% (91)	12.0% (129)	14.3% (175)	4.8% (12)
** Mean (SD)**													
**Age**	13.0 (4.4)	13.1 (3.8)	12.9 (4.6)	12.8 (4.3)	12.9 (4.3)	13.1 (3.7)	12.9 (4.3)	12.8 (4.3)	13.0 (4.0)	13.2 (3.9)	13.0 (4.8)	12.9 (4.4)	12.7 (4.6)
**Height (cm)**	157.0 (21.1)	158.0 (19.2)	157.7 (21.9)	154.2 (20.5)	153.5 (20.3)	160.2 (21.1)	159.7 (23.4)	156.4 (22.6)	155.8 (21.1)	155.1 (15.5)	155.2 (18.2)	151.3 (16.5)	150.1 (17.9)
**Weight (kg)**	55.4 (27.1)	55.4 (23.9)	58.8 (31.4)	54.6 (27.2)	50.8 (24.5)	56.8 (25.5)	58.5 (30.9)	56.1 (29.1)	53.1 (26.3)	53.4 (21.2)	59.1 (31.4)	52.6 (24.4)	47.5 (20.5)
**DXA variable**s													
** Total body aBMD**	0.98 (0.20)	0.98 (0.18)	1.03 (0.23)	0.96 (0.20)	0.96 (0.19)	0.99 (0.19)	1.03 (0.23)	0.97 (0.21)	0.96 (0.19)	0.97 (0.17)	1.03 (0.22)	0.96 (0.19)	0.95 (0.19)
** Pelvic aBMD**	1.10 (0.32)	1.10 (0.28)	1.17 (0.37)	1.08 (0.31)	1.05 (0.31)	1.09 (0.29)	1.15 (0.37)	1.06 (0.33)	1.05 (0.32)	1.12 (0.26)	1.19 (0.35)	1.10 (0.29)	1.06 (0.29)
** Lumbar spine aBMD**	0.86 (0.25)	0.86 (0.22)	0.91 (0.28)	0.84 (0.24)	0.84 (0.23)	0.84 (0.22)	0.88 (0.27)	0.81 (0.24)	0.82 (0.22)	0.90 (0.22)	0.96 (0.29)	0.88 (0.23)	0.88 (0.24)
** Total percent fat**	29.2 (11.0)	28.9 (9.7)	28.6 (12.8)	30.9 (11.4)	28.4 (9.6)	25.8 (9.1)	24.8 (11.3)	27.8 (11.3)	25.9 (9.4)	33.0 (8.0)	33.5 (10.8)	34.9 (8.8)	32.2 (7.5)
** Total fat mass (kg)**	16.8 (12.7)	16.6 (11.0)	17.8 (15.9)	17.6 (13.2)	14.9 (10.1)	15.2 (10.3)	15.2 (13.6)	16.3 (13.0)	14.3 (10.3)	18.6 (11.4)	21.1 (17.4)	19.3 (13.1)	15.9 (9.6)
** Total lean mass (kg)**	37.3 (17.3)	37.5 (15.5)	39.6 (19.1)	35.9 (16.9)	34.8 (16.4)	40.3 (17.4)	41.9 (20.6)	38.6 (18.9)	37.7 (17.8)	33.7 (10.7)	36.7 (15.1)	32.2 (12.0)	30.5 (11.5)

*The “Other race” category includes: all non-Hispanic participants who reported a race other than White or African American, all who reported multiple races and the non-Hispanic Asians.

When we compared included and excluded participants in the sample, individuals included in our sample were younger (13.0 vs. 13.4 years, *p* = 0.002), heavier (underweight 2.7% vs. 7.2%, normal weight 61.5% vs. 59.1%, overweight 17% vs. 10.0%, and obesity 18.8% vs. 13.7%; *p* = 0.001), and had a lower proportion of minority children (White 62.2% vs. 54.7%, African American 13.6% vs. 23.1%, Hispanic 17.8% vs. 15.1%, and other 6.5% vs. 7.1%; *p* = 0.001).

The demographic characteristics of the sample are shown in Table 1. The average age was 13 years, and 17% and 18.8% of subjects had overweight and obesity, respectively. A higher proportion of African American and Hispanic children had overweight or obesity compared with White children. Mean aBMD was higher for African American children compared with White and Hispanic children.

### Total body aBMD

For total body aBMD, we found significant 2-way interactions for percent body fat (percent body fat x gender; percent body fat x race/ethnicity) (Table 2) and total fat mass (total fat mass (kg) x gender; total fat mass (kg) x race/ethnicity) (Table 3).

**Table 2:** Models of percent body fat and bone mineral density (aBMD) by region. 2-way interaction models of percent body fat with control variables of lean mass, gender, and race on outcomes of total, pelvic and lumbar spine aBMD.

	Total body aBMD			Pelvic aBMD			Lumbar spine aBMD		
	Unstandardized coefficients (B)	SE	p-value	Unstandardized coefficients (B).	SE	p-value	Unstandardized coefficients (B)	SE	p-value
**Model 1**									
% body fat	-0.0030	0.0002	<0.0001	0.0003	0.0003	0.3960	-0.0046	0.0004	< 0.0001
Lean mass (in kg)	0.0068	0.0001	<0.0001	0.0136	0.0003	<0.0001	0.0071	0.0002	<0.0001
White	Ref								
African American	0.0310	0.0083	<0.0001	0.0611	0.0118	<0.0001	0.0343	0.0115	0.0040
Hispanic	-0.0042	0.0089	0.6390	-0.0045	0.0133	0.7340	-0.0044	0.0119	0.7150
Other	-0.0490	0.0136	0.0010	-0.0826	0.0247	0.0010	-0.0523	0.0221	0.0220
AA * %body fat	0.0001	0.0002	0.0002	-0.0008	0.0004	0.0220	0.0001	0.0004	0.8270
Hispanic * %body fat	0.0002	0.0003	0.0003	0.0002	0.0004	0.7140	0.0003	0.0004	0.4970
Other * %body fat	0.0017	0.0005	<0.0001	0.0027	0.0008	0.0020	0.0021	0.0008	0.0090
Male	Ref								
Female	0.0128	0.0081	0.1180	0.0902	0.0140	<0.0001	0.0816	0.0148	<0.0001
Female* %body fat	0.0012	0.0003	<0.0001	0.0007	0.0004	0.1080	0.0018	0.0004	<0.0001
Age	0.0172	0.0005	<0.0001	0.0146	0.0009	<0.0001	0.0215	0.0008	<0.0001

**Table 3:** Models of total fat mass and bone mineral density (aBMD) by region. 2-way interaction models of total fat mass (in kg) with control variables of lean mass, gender, and race/ethnicity on outcomes of total body and pelvic aBMD.

	Total body aBMD			Pelvic aBMD		
	Unstandardized coefficients (B)	SE	p-value	Unstandardized coefficients (B)	SE	p-value
**Model 2**						
Fat mass kg	-0.0044	0.0003	<0.0001	0.0143	0.0003	< 0.0001
Lean mass kg	0.0085	0.0002	<0.0001	-0.0019	0.0004	<0.0001
White	Ref					
African American	0.0307	0.0049	<0.0001	0.0359	0.0069	<0.0001
Hispanic	-0.0042	0.0056	0.458	-0.0051	0.0086	0.5590
Other	-0.0162	0.0084	0.0600	-0.0523	0.0148	0.0010
AA* Fat mass (kg)	0.0002	0.0002	0.3870	-0.0001	0.0003	0.8020
Hispanic * Fat mass (kg)	0.0004	0.0002	0.0002	0.0004	0.0004	0.3140
Other* Fat mass (kg)	0.0012	0.0005	0.0180	0.0032	0.0010	0.0010
Male	Ref					
Female	0.0030	0.0037	0.4310	0.0706	0.0068	<0.0001
Female* Fat mass (kg)	0.0029	0.0002	<0.0001	0.0030	0.0004	<0.0001
Age	0.0158	0.0005	<0.0001	0.0125	0.0009	<0.0001

**Fig 1:**
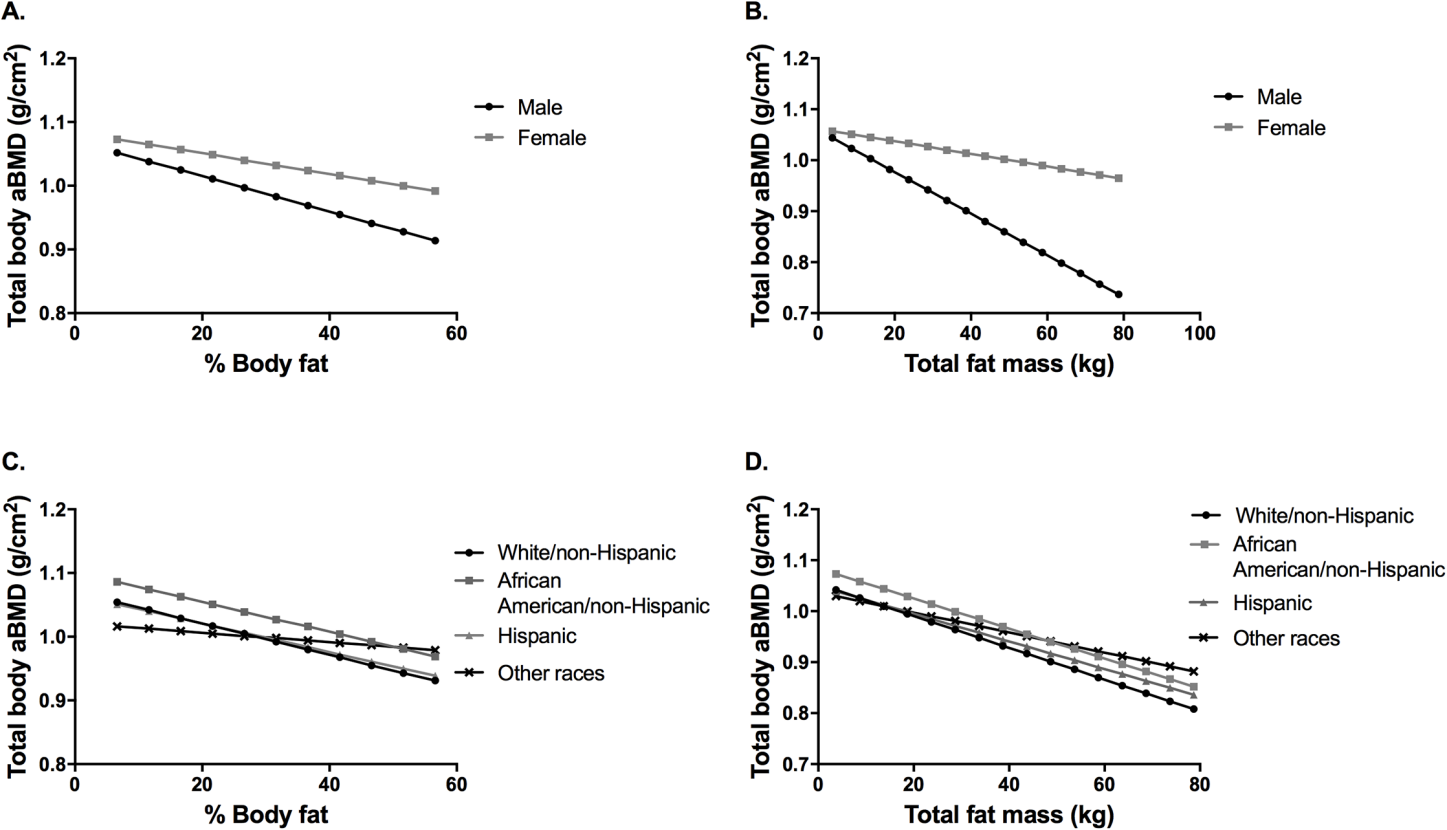
Models of adiposity on total body bone mineral density (aBMD). Graphical display of effect of sex interaction (A and B) and race/ethnicity interaction (C and D) fixing all other predictors at their mean.

Fig 1 displays predicted values of total body aBMD according to gender and race. Overall there is a trend of lower total body aBMD with increasing percent body fat (Fig 1A) and increasing total fat mass (Fig 1B), with different slopes for males versus females and for the different racial groups (Fig 1C and Fig 1D). Males have a steeper rate of decrease in total body aBMD than females as percent body fat and total fat mass increase.

### Pelvic aBMD

**Fig 2:**
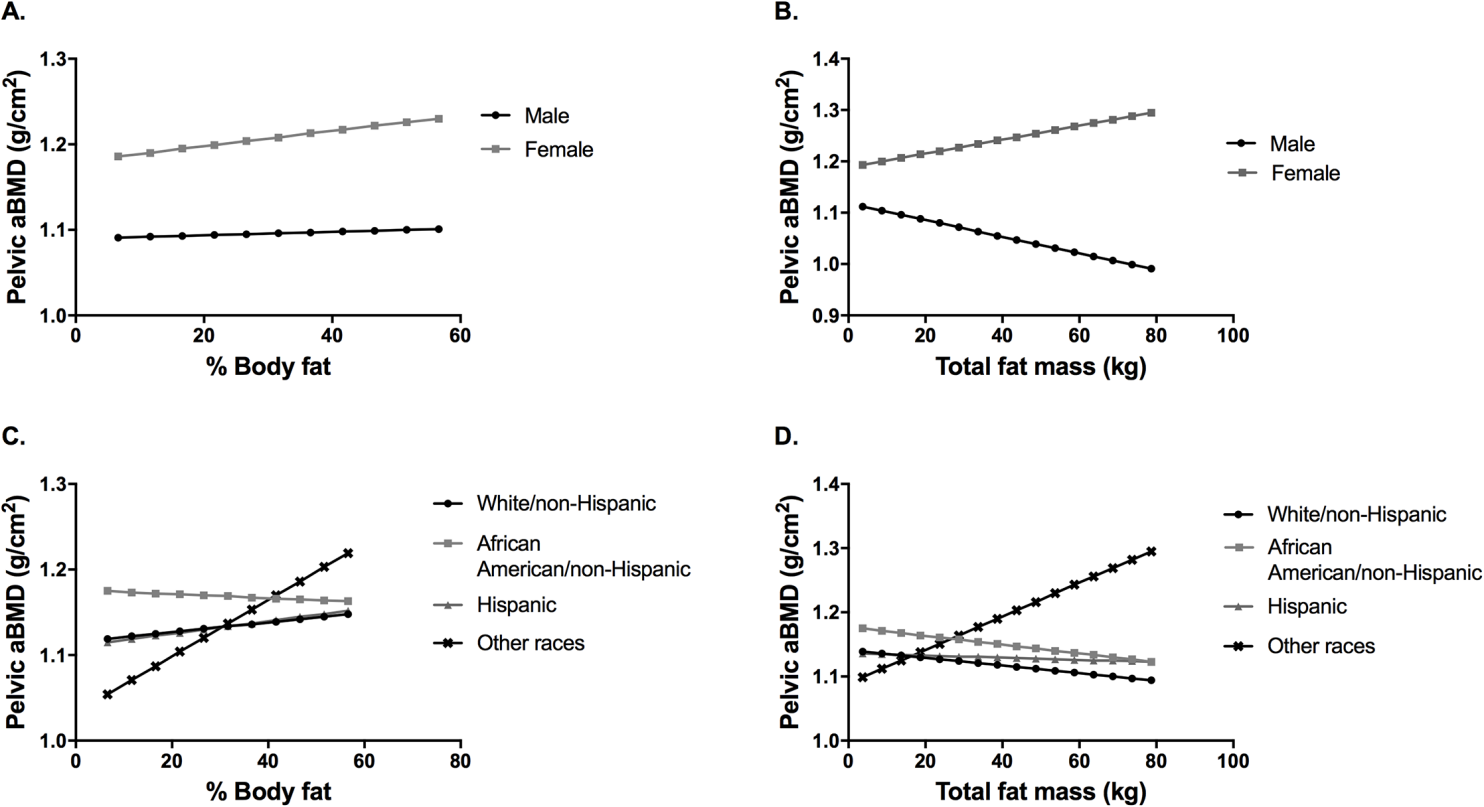
Models of adiposity on pelvic bone mineral density (aBMD). Graphical display of effect of sex interaction (A and B) and race/ethnicity interaction (C and D) fixing all other predictors at their mean.

For pelvic aBMD, we found significant 2-way interactions for percent body fat (percent body fat x gender; percent body fat x race/ethnicity) (Table 2) and total fat mass (total fat mass (kg) x gender; total fat mass (kg) x race/ethnicity) (Table 3). Fig 2 displays predicted values of pelvic aBMD according to gender and race. With increasing percent body fat, there was a trend of higher pelvic aBMD for males and females (Fig 2A). With increasing total fat mass, there was a trend of higher pelvic aBMD for females, but lower pelvic aBMD for males (Fig 2B). Relationships were also mixed for the different races. With increasing percent body fat, White, Hispanic and Other races showed an increasing trend of pelvic aBMD, while it decreased in African Americans (Fig 2C). With increasing total fat mass, the pelvic aBMD decreased across races, except for the other race group (Fig 2D).

### Lumbar spine aBMD

For lumbar spine aBMD, we only found significant 2-way interactions for the percent body fat model (percent body fat x gender; percent body fat x race/ethnicity) (Table 2) and the 3-way interaction model for total fat mass (total fat mass (kg) x gender x race/ethnicity) (Table 4).

**Table 4:** Model of total fat mass and lumbar spine bone mineral density (aBMD). 3-way interaction model of total fat mass (kg) with control variables of lean mass, gender, and race/ethnicity on outcomes of lumbar spine aBMD.

	Lumbar spine aBMD		
	Unstandardized coefficients (B)	SE	P-value
**Model 2**			
Fat mass kg	-0.0066	0.0004	<0.0001
Lean mass kg	0.0096	0.0003	<0.0001
White	Ref.		
African American	0.0180	0.0079	0.0250
Hispanic	-0.0102	0.0077	0.1920
Other	-0.0027	0.0176	0.8770
African-American * Female	0.0349	0.0144	0.0190
Hispanic * Female	0.0252	0.0147	0.0930
Other * Female	-0.0194	0.0244	0.4290
Male	Ref.		
Female	0.0560	0.0090	<0.0001
Female * Fat mass (kg)	0.0049	0.0006	<0.0001
AA * Fat mass (kg)	0.0010	0.0005	0.0570
Hispanic * Fat mass (kg)	0.0009	0.0004	0.0600
Other * Fat mass (kg)	0.0005	0.0010	0.6660
Female * AA * Fat mass (kg)	-0.0017	0.0008	0.0400
Female * Hispanic * Fat mass (kg)	-0.0014	0.0007	0.0620
Female * Other * Fat mass (kg)	0.0019	0.0016	0.2470
Age	0.0196	0.0008	<0.0001

**Fig 3:**
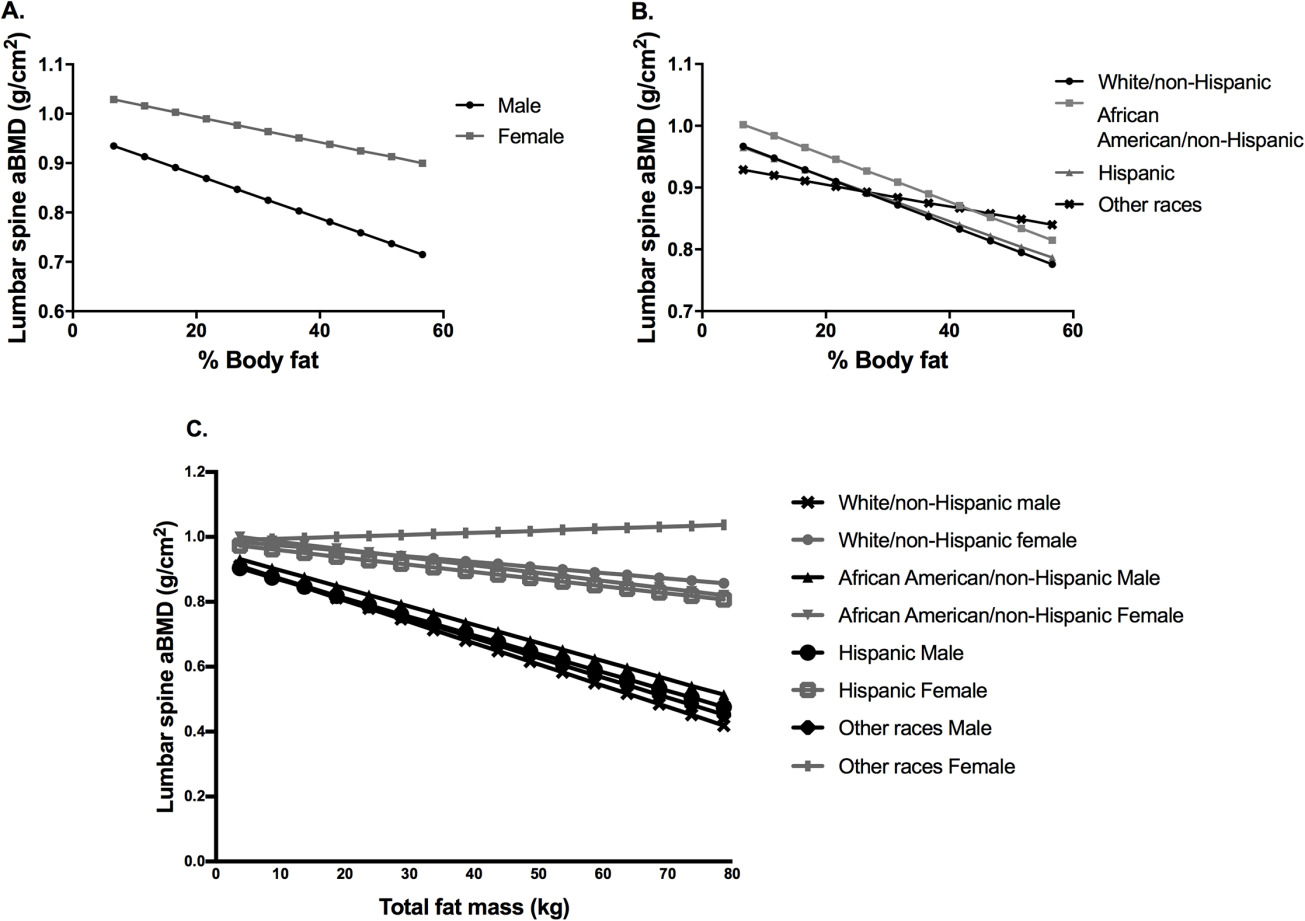
Models of adiposity on lumbar spine bone mineral density (aBMD). Graphical display of effect of sex interaction (A) and race/ethnicity interaction (B) 3-way interaction model for total fat mass (kg) with lumbar spine aBMD (C).

Fig 3 displays predicted values of lumbar spine aBMD according to gender and race/ethnicity. With increasing percent body fat, lumbar spine aBMD decreased in both genders (Fig 3A) and races (Fig 3B). Females have a higher average lumbar spine aBMD than males. African-Americans have the highest predicted lumbar spine aBMD followed by Whites and Hispanics. With increasing total fat mass, lumbar spine aBMD decreased for all categories except for females of other race/ethnicity (Fig 3C).

## Discussion

In our study, we found significant and novel interactions by gender and race/ethnicity in the associations between percent body fat and total fat mass (kg) with total body, pelvic and lumbar spine aBMD. Both total body aBMD and lumbar spine aBMD decreased with increasing percent body fat and total fat mass in both genders and all races. We found less consistent patterns for pelvic aBMD, which increased in both genders as percent body fat increased, but showed this positive trend with increasing total fat mass in females and the opposite in males.

Overall, females had higher total body, pelvic and lumbar spine aBMD than males in our models. These gender differences could be multifactorial: different pubertal status of participants, gender differences in body composition (with females having more fat mass than males, and males having higher lean mass), hormonal environment, and the effect of childhood obesity on puberty onset. While this may be dependent on pubertal status one of the limitations in this study is the lack of pubertal status in the database. However, another factor that might be leading to gender differences in our results is a difference in adipose tissue distribution. It has been well described that adipose tissue distribution is predominantly subcutaneous in females and more visceral central adiposity in males. This central adiposity is directly correlated with obesity-associated comorbidities, such as metabolic syndrome, dyslipidemia, insulin resistance and a low-grade inflammation which may affect the relationship between bone and adipose tissue in different ways [23]. A higher percent body fat has been associated with increased bone mineral density in girls during puberty and may be explained by these differences in adiposity during puberty [24]

Race differences in bone density have also been previously described with studies reporting that African American and Hispanic children have higher BMDs compared to Caucasian children [10], while others report a greater aBMD for African Americans compared to Caucasians, and Caucasians having a greater aBMD than Asian and Hispanics. [25] The genetic factors that determine bone size and mineralization are responsible for the majority of these racial differences [25]. As with gender, differences in lean mass by race may also explain the variable relationship of race on bone density [15] which is a reason these models adjusting for lean mass and still demonstrating a significant race difference are important.

The relationship between adiposity and bone mineral density (BMD) in the pediatric population is not completely understood, and the literature is quite mixed. Some studies have found negative associations between percent body fat and lumbar spine BMD [26] and whole body BMC [27] in females between 9 to 24 years of age (n = 521). Other studies have reported positive associations between fat mass and lean mass with total, lumbar spine and pelvic BMD in young premenopausal women aged 20 to 25 (n = 921) [28]. The race and sex interactions may account for some of the inconsistent findings of associations reported in the literature. A recent systematic review and meta-analysis found: 1) moderate quality of evidence for a higher total BMD in children with obesity when compared with those who were overweight, 2) moderate quality of evidence for a significant mean difference of lumbar spine BMD in the normal–weight group compared to the overweight or obese groups, and 3) no significant difference in femoral neck BMD in children with normal-weight when compared with those who were overweight. [29] They report significant statistical heterogeneity in all the results described above.[29]

Given the mixed results in the current literature our study using direct measures of adiposity (both percent body fat and total fat mass), lean mass, and areal bone mineral density (aBMD) using DXA across multiple regions of the body clarifies many of the results in prior studies. In addition, our study uses a nationally representative sample with a large number of participants and ethnic diversity with oversampling of minority children. Limitations of our study include the cross-sectional study design, which does not prove causality, the lack of information about long-term clinical outcomes like fractures, and a lack of information on clinical parameters such as pubertal status, hormone measurements, vitamin D levels, or physical activity. We also acknowledge that the children from our study population were younger, heavier, and had a lower proportion of minority children. Additional limitations due to what was included in this survey are the regions of spine and pelvis that are taken from whole body scans and hence may not be as accurate as focused DXA scans or volumetric BMD measured by Quantitative CT (QCT) scans. As recommended by the International Society For Clinical Densitometry, the use of DXA in children (≥3 years of age) and adolescents is the preferred method and it is well accepted due to the accurate and precise BMC and aBMD provided [30] and the low radiation exposure. [31] DXA is limited because it measures areal BMD instead of true volumetric BMD which may be important when the skeleton is still growing, and bone size might be influencing BMD measures [31]. Quantitative computerized tomography (QCT) measures bone size, shape and true volumetric BMD (vBMD) but are not yet widely available. [30, 32] Both negative [33, 34] and positive [35] associations between fat mass and cortical bone of the tibia, and a negative association between fat mass and the radius[36] have been reported with this technique. In addition, with the use of DXA, analysis can be performed in two regions along with the use of whole body BMD. It is possible given the lower ages of some of the participants that the total body less head (TBLH) variable could have yielded different results but analysis showed that there was a strong correlations between these two factors.

While the findings in our study overall suggest that obesity has a negative impact on total and lumbar aBMD, weight bearing regions such as the pelvis are possibly spared from the impact of adiposity on bone health. Future prospective studies are needed to clarify the impact of adiposity on bone density and quality in the pediatric population, using both DXA and QCT methods. Gender, race/ethnicity and pubertal status can also influence this association and it is crucial to understand this differences when interpreting DXA scans. While this retrospective clinical trial cannot directly evaluate mechanisms for the findings in the current study, pre-clinical investigations suggest that adiposity may directly correlate with marrow adiposity which is associated with a decline in bone density and secondary bone fragility. [37]

If childhood obesity is limiting peak bone accrual and increasing the risk for osteoporosis and/or fractures later in life, pediatricians can evaluate bone mineral density in children who have expanded adipose tissue. If lower BMD than expected is detected, some interventions like increasing physical activity, treating vitamin D and nutritional deficiencies can improve bone health during this critical period significantly. A close follow-up and the report of clinical outcomes of these children can also help close the gap existing in the literature.

## Abbreviations

NHANES: National Health Examination Survey
BMD: Bone Mineral density
aBMD: areal Bone Mineral Density
BMC: Bone mineral content
DEXA: Dual Energy X-ray Absorptiometry
